# Met Is the Most Frequently Amplified Gene in Endometriosis-Associated Ovarian Clear Cell Adenocarcinoma and Correlates with Worsened Prognosis

**DOI:** 10.1371/journal.pone.0057724

**Published:** 2013-03-04

**Authors:** Yoriko Yamashita, Shinya Akatsuka, Kanako Shinjo, Yasushi Yatabe, Hiroharu Kobayashi, Hiroshi Seko, Hiroaki Kajiyama, Fumitaka Kikkawa, Takashi Takahashi, Shinya Toyokuni

**Affiliations:** 1 Department of Pathology and Biological Responses, Nagoya University Graduate School of Medicine, Nagoya, Aichi, Japan; 2 Department of Pathology, Nagoya City University Hospital, Nagoya, Aichi, Japan; 3 Department of Obstetrics and Gynecology, Nagoya University Graduate School of Medicine, Nagoya, Aichi, Japan; 4 Department of Pathology and Molecular Diagnostics, Aichi Cancer Center Hospital, Nagoya, Aichi, Japan; 5 Division of Molecular Carcinogenesis, Center for Neurological Diseases and Cancer, Nagoya University Graduate School of Medicine, Nagoya, Aichi, Japan; Institute for Virus Research, Laboratory of Infection and Prevention, Japan

## Abstract

Clear cell adenocarcinoma of the ovary (OCC) is a chemo-resistant tumor with a relatively poor prognosis and is frequently associated with endometriosis. Although it is assumed that oxidative stress plays some role in the malignant transformation of this tumor, the characteristic molecular events leading to carcinogenesis remain unknown. In this study, an array-based comparative genomic hybridization (CGH) analysis revealed Met gene amplification in 4/13 OCC primary tumors and 2/8 OCC cell lines. Amplification of the AKT2 gene, which is a downstream component of the Met/PI3K signaling pathway, was also observed in 5/21 samples by array-based CGH analysis. In one patient, both the Met and AKT2 genes were amplified. These findings were confirmed using fluorescence *in situ* hybridization, real-time quantitative PCR, immunoblotting, and immunohistochemistry. In total, 73 OCC cases were evaluated using real-time quantitative PCR; 37.0% demonstrated Met gene amplification (>4 copies), and 8.2% had AKT2 amplification. Furthermore, stage 1 and 2 patients with Met gene amplification had significantly worse survival than patients without Met gene amplification (p<0.05). Met knockdown by shRNA resulted in reduced viability of OCC cells with Met amplification due to increased apoptosis and cellular senescence, suggesting that the Met signaling pathway plays an important role in OCC carcinogenesis. Thus, we believe that targeted inhibition of the Met pathway may be a promising treatment for OCC.

## Introduction

Clear cell adenocarcinoma of the ovary (OCC) is frequently associated with endometriosis [1], and the existence of abundant free iron in endometriotic cysts due to hemorrhage is proposed as a cause of persistent oxidative stress and subsequent carcinogenesis [2]. Oxidative stress due to iron overload causes genomic amplification in ferric nitrilotriacetate (Fe-NTA)-induced rat carcinoma cells [3], and the genomic changes observed in these animals are specific, showing close similarity to human tumors [4]. OCC is a chemo-resistant tumor with a relatively poor prognosis [5], and recent reports suggest that specific molecular events such as an activating mutation of the alpha-catalytic domain of PI3 kinase (PI3K) [6] or an inactivating mutation of AT-rich interactive domain 1A (ARID1A) [7], [8] may play roles in the tumorigenesis of OCC. However, focusing on genomic copy number change analyses, multiple studies performed by different groups using either comparative genomic hybridization (CGH) or array-based CGH analysis in OCC cases have failed to demonstrate specific gene amplification [9]–[11]. Recently, a study from the United Kingdom reported Her2 amplification at chromosome 17q12 in 14% of the investigated OCC cases using array-based CGH analysis [12], emphasizing the molecular heterogeneity of the tumor. Using double in situ hybridization (DISH) and immunohistochemistry, Yamamoto et al also reported Met amplification in 28% of Japanese OCC cases [13]. Most recently, another report from Japan demonstrated that ZNF217 at chromosome 20q13.2 was amplified in 20% of OCC patients [14]. In this study, we performed an array-based CGH analysis using Japanese OCC samples and detected genomic amplification of the Met gene in 6/21 samples. Additionally, we determined that the Met gene was the most frequently amplified gene in these samples. We also detected amplification of the AKT2 gene, which is one of the three isoforms of AKT kinase, a downstream component of the Met/PI3K signaling pathway. This is the first study to report the frequent amplification of a specific gene in OCC detected by array-based CGH analysis and the first to report AKT2 amplification in OCC. We further analyzed a larger number of OCC samples in knockdown experiments to investigate the role of the Met/PI3K/AKT pathway in OCC tumorigenesis.

## Materials and Methods

### Patients and Samples

Formalin-fixed, paraffin-embedded tissues from 73 ovarian clear cell carcinoma patients and 3 ovarian endometrial adenocarcinoma patients at Nagoya University Hospital were obtained with written informed consent. Microscopically negative lymph node samples without metastasis were also obtained from the patients for use as controls. The experimental designs of the genomic and expression studies were reviewed and approved by the Committee for Bioethics of Nagoya University Graduate School of Medicine (#671).

### Cell Lines

ES-2, KOC-7C, RMG-II, and TOV21G were cultured with RPMI-1640 (Sigma) with 10% FBS. JHOC-5, JHOC-7, JHOC-8, and JHOC-9 cells were provided from Riken BRC, Tsukuba, Japan, and were cultured with DMEM/F12 (Sigma)-based medium, according to the distributor’s instructions.

### Array-based Comparative Genomic Hybridization

Genomic DNA was isolated and labeled using the Oligonucleotide Array-Based CGH for Genomic DNA Analysis (ULS labeling) Kit (Agilent Technologies, Santa Clara, CA, USA), according to the manufacturer’s instructions. Briefly, 4 continuous 5 µm paraffin-sections were placed in an Eppendorf tube, and after paraffin removal and proteinase K treatment, genomic DNA was extracted using the DNeasy Blood & Tissue Kit (Qiagen, Valencia, CA, USA) with modifications. After 5 minutes of heat fragmentation at 95°C, reference DNA from the lymph node samples was labeled with Cy3, and tumor DNA was labeled with Cy5. The two samples were then mixed together after the removal of residual unlabeled fluorescent dye and then hybridized to a Human Genome CGH 244A Oligo Microarray (G4411B, Agilent Technologies). After washing, stabilization, and drying, the microarrays were scanned with an Agilent Scanner (Agilent) and analyzed with DNA Analytics Software (ver. 4.0) (Agilent). Genomic DNA was obtained from cell lines and control early passage immortalized human female B cells [15] for copy number reference and then applied to the array-based CGH analysis as previously described [16].

### Fluorescence *In Situ* Hybridization

Bacterial artificial chromosome clones (RP11-95I20 and RP11-163C9 for the Met gene) were selected from http://genome.ucsc.edu/and purchased from http://bacpac.chori.org/. DNA was then extracted by the NucleoBond PC 20 Plasmid DNA Purification Kit (Macherey-Nagel, Düren, Germany) with modifications.

Fluorescent probes were labeled by incorporating Green-dUTP (Vysis; Abbott Laboratories; Abbott Park, IL, USA) into the DNA using the Nick Translation Kit (Vysis). CEP 7 (D7Z1) Spectrum Orange Probe (Vysis) was purchased as a control probe. Fluorescence *in situ* hybridization (FISH) was performed using the probes, Paraffin Pretreatment Kit, and LSI/WCP Hybridization Buffer (Vysis) according to the manufacturer’s protocol. Briefly, paraffin sections were treated with protease, and after denaturation, the probes were hybridized to nuclear DNA, counterstained with DAPI, and visualized using a fluorescence microscope.

### Real-time Quantitative PCR

A total of 50 ng of isolated genomic DNA was applied per reaction for real-time quantitative PCR (qPCR). Sample DNA was amplified for 40 cycles with an annealing temperature of 55°C using the TaqMan Universal PCR Kit (ABI) and 7300 Real-time PCR System (ABI) following the manufacturer’s instructions. The sequences of the primers and internal probes were as follows: Met (sense), 5′-TCCTGGGCACCGAAAGG-3′; Met (antisense), 5′-GAGGCGAGGGATTGGGTACT-3′; Met reporter probe,

5′-FAM-CAGCCCCTTTCAGATC-MGB-3′. For the AKT2 gene, a TaqMan Copy Number Assay Kit for AKT2 (Hs00113634; ABI) including the FAM/MGB-labeled AKT2 probe was used. VIC-TAMRA-labeled TaqMan Copy Number Reference (TERT) primers and probes (ABI) were used as controls for both assays. PCR products were quantified in triplicate and normalized to the human TERT gene using the Delta-delta Ct method according to the manufacturer. To compare the relative amount of AKT isoform expression, a quantitative reverse transcription PCR (qRT-PCR) analysis was performed using plasmids encoding the PCR products of AKT1 or AKT2 using the following primers: AKT1 (sense) 5′-CCCAAGCACGCGTGACCAT-3′; AKT1 (antisense) 5′-GCGTAGTAGCGGCCTGTGGC-3′; AKT2 (sense) 5′-GAGGTCATGGAGCACAGGTT-3′; AKT2 (antisense) 5′-CTGGTCCAGCTCCAGTAAGC-3′.

PCR products (100–200 bp) were subcloned into the pGEMTEasy vector (Promega, Madison, WI) by TA-cloning according to the manufacturer’s instructions, and the plasmids were then used as control templates to establish standard curves. Then, qRT-PCR analyses were performed to detect AKT1 or AKT2 as previously described [17].

### Immunoblotting and Immunohistochemistry

The primary antibodies against human c-Met, AKT1, AKT2, and phosphorylated-AKT (Ser473) were clones 3D4 (Invitrogen, Carlsbad, CA, USA), D26, C7H310, D6G4, and 736E11 (Cell Signaling Technologies, Danvers, MA, USA), respectively. Western blotting and immunohistochemical staining were performed as previously described [15], [17]. The established scoring criteria were used for immunohistochemical staining as follows:

2+: more than 50% positive tumor cells with strong intensity in the cell membrane.

1+: less than 50% positive tumor cells with strong intensity in the cell membrane or more than 50% but weak staining in the tumor cell membrane or any intensity staining without tumor cell membrane staining.

- : no staining.

### Retroviral and Lentiviral Transduction of Short-hairpin RNA

The construction of short-hairpin RNA (shRNA)-encoding retroviral vectors were performed as previously described [15]. Briefly, the precursor form of shRNA was ligated into an entry vector encoding the human H1 promoter and then transferred to the destination vectors by the Gateway system (Invitrogen). The lentiviral vectors pCS-RfA-CG, pCAG-HIVgp, and pCMV-VSV-G-RSV-Rev were obtained from Dr. Miyoshi’s lab (Riken BRC, Tsukuba, Japan). After transfer of the H1 promoter and shRNA precursor, shRNA-encoding lentiviruses were produced by the transient transfection of 3 plasmids into 293T cells, and the harvested viral particles were subsequently used for target cell transduction in nearly the same manner as the retroviruses described above. The sense-coding sequences of the shRNA were as follows: shMet 2, GCAGTGAATTAGTTCGCTA; shMet5, GGGAATCATCATGAAAGAT; shNC, ATCTGAAGACCTATTTTAT.

### Cell Growth and Survival Assays

OCC cell lines (JHOC-5, JHOC-8, and ES-2) and control HeLa cells were transiently transfected by Met-shRNA- or control shRNA-coding retroviral vectors described above. Cells were transferred to chamber slides after 24 hours. At day 2 post-transfection, cells were fixed with neutralized 4% formalin and then subjected to a TdT-mediated dUTP-biotin nick end labeling (TUNEL) assay and a senescence assay using the ApopTag Plus Peroxidase In Situ Apoptosis Detection Kit (Chemicon International, Temecula CA, USA) and Senescence Detection Kit (BioVision Research Products, Mountain View, CA, USA), respectively. To estimate the numbers of viable cells, cells were transferred to 96-well plates 24 hours after transfection. At day 4 post-transfection, 250 µl of 0.4 mg/ml thiazolyl blue tetrazolium bromide (MTT) was added to each well and incubated for 1 hour at 37°C. After removal of the solution, 100 µl of dimethylsulfoxide was added to each well and incubated for 10 minutes at room temperature. Finally, the absorbance due to formazan formation was determined at 570 nm.

### Statistical Analyses

Student’s t-test was used to analyze the MTT assay results. A Kaplan-Meier analysis was performed with the log-rank test using SPSS (IBM) software version 17.0.

## Results

### Array-based Comparative Genomic Hybridization Analysis

**Figure 1:**
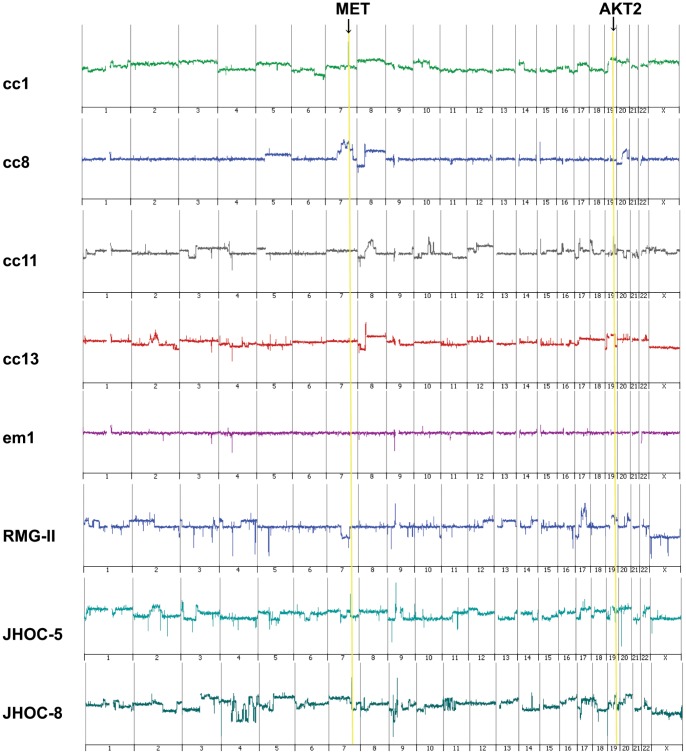
Representative array-based CGH analysis data of chromosomes 1–22 and X. The data of 4 ovarian clear cell adenocarcinoma samples (cc1, cc8, cc11, cc13), 1 endometrioid adenocarcinoma sample (em1), and 3 cell lines (RMG-II, JHOC-5, and JHOC-8) are shown. Met amplification is observed in cc1, cc8, and cc13 and JHOC-5 and JHOC-8 cells. AKT2 amplification is observed in cc11 and cc13 and JHOC-8 and RMG-II cells.

Of the 73 OCC cases, DNA extracted from 13 tumors and 3 endometrioid adenocarcinoma samples was subjected to array-based CGH analysis. The genomic gains of the chromosome 7q31.31 region were frequently less than 2 Mb in length, showing peaks at the area of the Met oncogene, and were detected in 4 of the 13 OCC cases (cc1, cc2, cc8, cc13) (**Figs. 1** **and** **2**). Frequent amplification of the 19q3.2 region including the AKT2 gene with a smaller peak (less than 1 Mb) was also observed in 3 of the 13 cases (cc5, cc11, cc13). One case (cc13) had amplification of both genes (i.e., Met and AKT2). We then performed an array-based CGH analysis of 8 OCC cell lines and detected amplification of the Met gene in 2: JHOC-5 and JHOC-8. The other 134 genomic regions amplified in more than 20% of the 21 samples, including 13 primary tumors and 8 cell lines, are shown in **Table S1**. The genes other than Met and AKT2 with previously known functions positively affecting cell growth were Lyn, P-Rex2, Jag2, Map3K10, and Bcl3 (shown in red in **Table S1**), but the significance of the genes included in other amplified segments remains unclear. No amplification or deletion was observed in the CDKN2A region of chromosome 9 or the PTEN-encoding region of chromosome 10. Met amplification was not observed in any of the three endometrioid adenocarcinoma samples, and the genomic changes were rather inconspicuous compared to the clear cell carcinoma samples (**Figs. 1** **and** **2**). Summarizing the array-based CGH results, the copy number aberration analysis revealed that chromosomes 8q, 19q, 1q, and 20q had a relatively high frequency of genome gain, and chromosome 8p, 19p, and 17p tended to have genome loss (**Fig. S1**). The clinical, histological, and genetic data of the 13 OCC cases and 3 endometrioid adenocarcinoma cases used in the array-based CGH analyses are summarized in **Table 1**.

**Figure 2:**
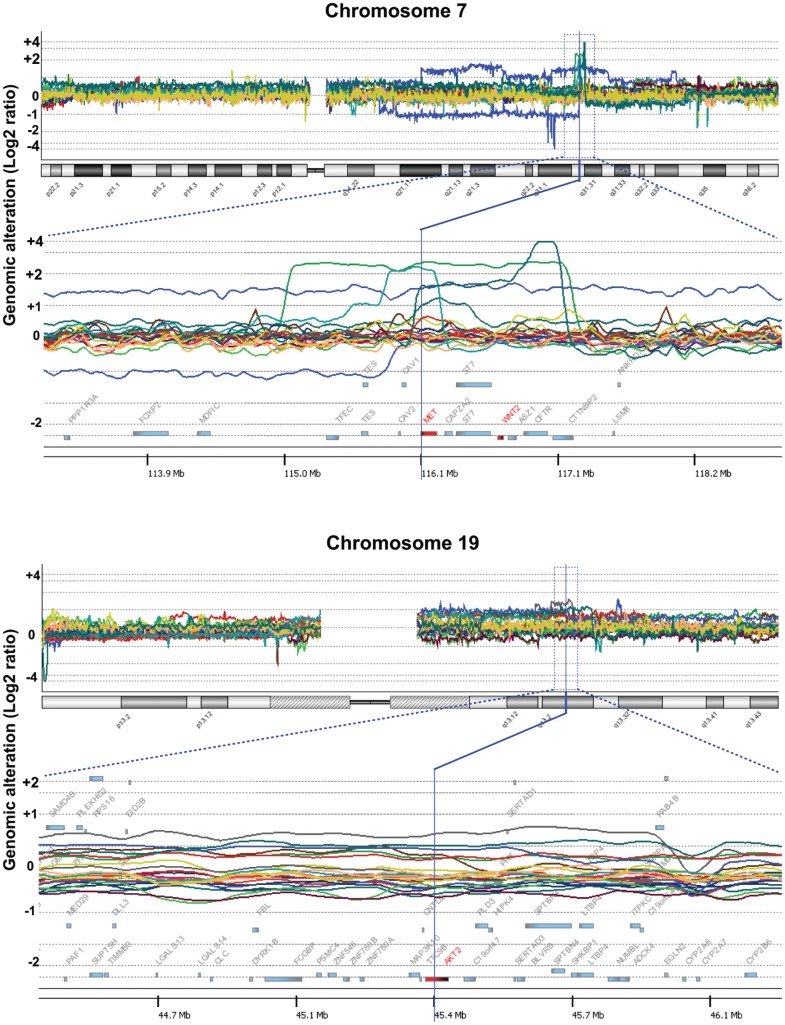
Gene and chromosome views of the amplified regions in array-based CGH analysis. Genomic changes in the 21 ovarian clear cell adenocarcinoma samples are shown. High, clear peaks within 2 Mb are observed in 6/21 samples at the genomic region encoding Met (upper row). The amplified region including AKT2 is also shown (lower row).

**Table 1:** Summary of the histological features and genomic changes in samples applied to the array-based comparative genomic hybridization.

	Histologic Features			Met Immunostaining	Met		AKT2		Other aCGH Characteristics	
Sample	Cysts	Hemorrhage or Hemosiderin Deposits	Endometriosis		aCGH	qPCR	aCGH	qPCR	p16 copy no.	PTEN copy no.
cc1	+	+	+	++	+++	5.4		<2	nc	nc
cc2	+	+	+	++	++	2.1		<2	nc	nc
cc3	+	+	+	+		<1.5		<2	nc	nc
cc4	+	+	−	+		<1.5		<2	nc	nc
cc5	+	+	+	+		<1.5	++	2.6	nc	nc
cc6	−	−	−	+		<1.5		<2	nc	nc
cc7	+	+	+	+		<1.5		<2	nc	nc
cc8	+	+	+	++	++	3.6		<2	nc	nc
cc9	+	+	+	+		<1.5		<2	nc	nc
cc10	+	+	+	+		<1.5		<2	nc	nc
cc11	+	+	+	+		<1.5	++	3.2	nc	nc
cc12	+	+	+	+		<1.5		<2	nc	nc
cc13	+	+	−	++	+	1.6	++	2.6	nc	nc
em1	+	+	−	−		<1.5		<2	nc	nc
em2	+	−	+	+		<1.5		<2	nc	nc
em3	+	−	−	−		<1.5		<2	nc	nc

aCGH: array-based comparative genomic hybridization, qPCR: real-time quantitative PCR, nc: no change.

### Confirmation of Met Amplification

**Figure 3:**
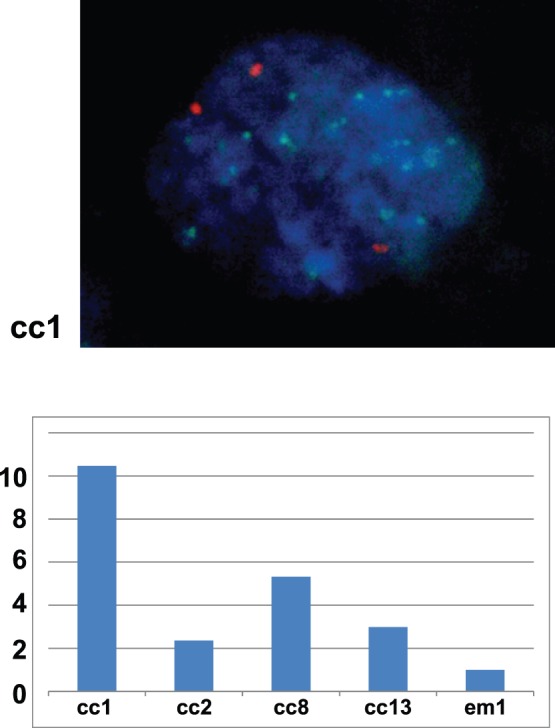
FISH analysis for confirmation of Met amplification. A representative nucleus of a Met-amplified cell (cc1) is shown in the upper figure (Green: Met probe, Orange: CEP 7; centromere 7 probe, Blue; DAPI). The lower graph shows the FISH signal number (MET/CEP 7 ratio) of the 4 Met-amplified ovarian clear cell adenocarcinoma samples (cc1, cc2, cc8, cc13) and an endometrioid adenocarcinoma case (em1) without Met amplification. A total of 60 cells were counted for each sample, average numbers (Met: CEP7) were as follows; cc1(18∶2.0), cc2(4.4∶2.1),cc8(10∶2.2), cc13(4.6/1.7), em1(1.7/1.9). All values were then normalized with that of em1 as 1.0.

**Figure 4:**
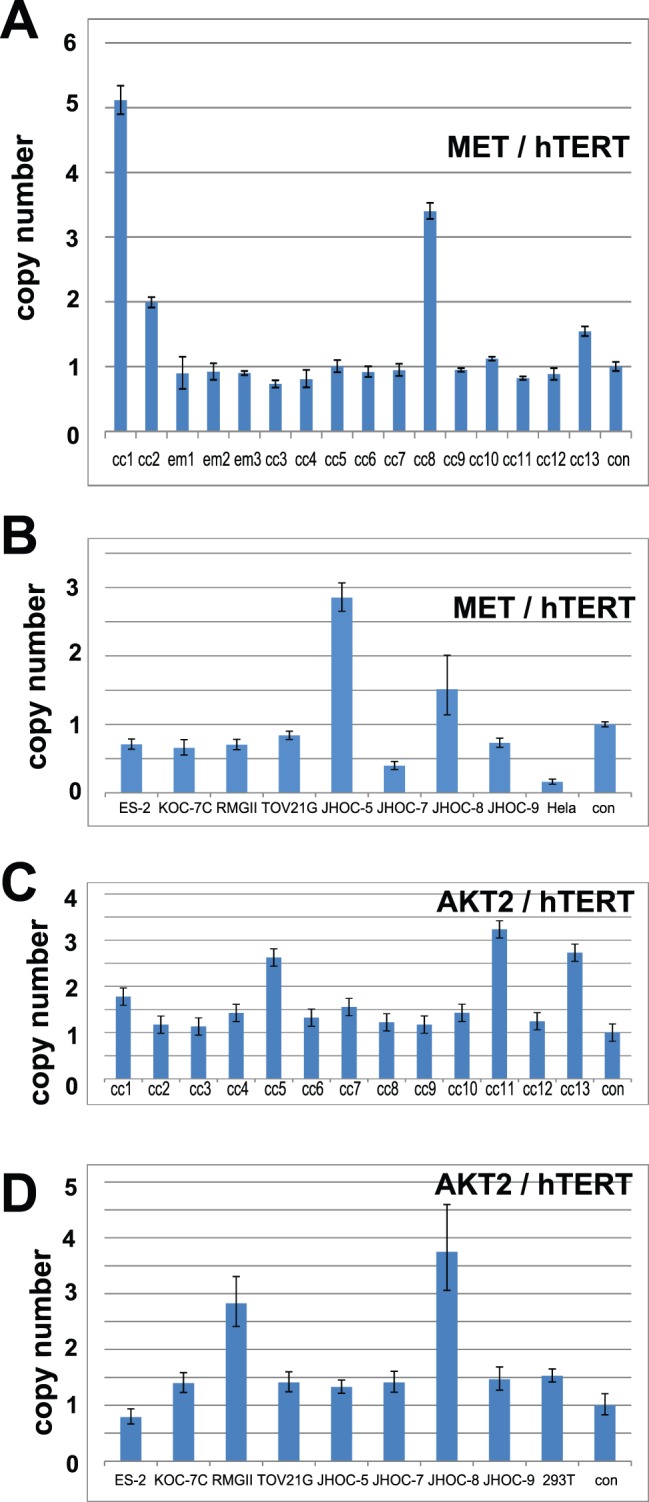
QPCR confirmation of Met and AKT2 amplification. **A.** Copy number change analysis of Met in 13 ovarian clear cell adenocarcinoma samples are shown. Four samples (cc1, cc2, cc8, cc13) had a Met/hTERT ratio greater than 1.5. **B.** Copy number change analysis of Met in 8 ovarian clear cell adenocarcinoma cell lines are shown. Two cell lines (JHOC-5 and JHOC-8) had a Met/hTERT ratio greater than 1.5. **C.** Copy number change analysis of AKT2 in 13 ovarian clear cell adenocarcinoma samples are shown. Three samples (cc5, cc11, cc13) had an AKT2/hTERT ratio greater than 1.5. **D.** The qPCR results for the copy number change analysis of AKT2 in 8 ovarian clear cell adenocarcinoma cell lines are shown. Two cell lines (RMG-II and JHOC-8) had a Met/hTERT ratio greater than 1.5.

**Figure 5:**
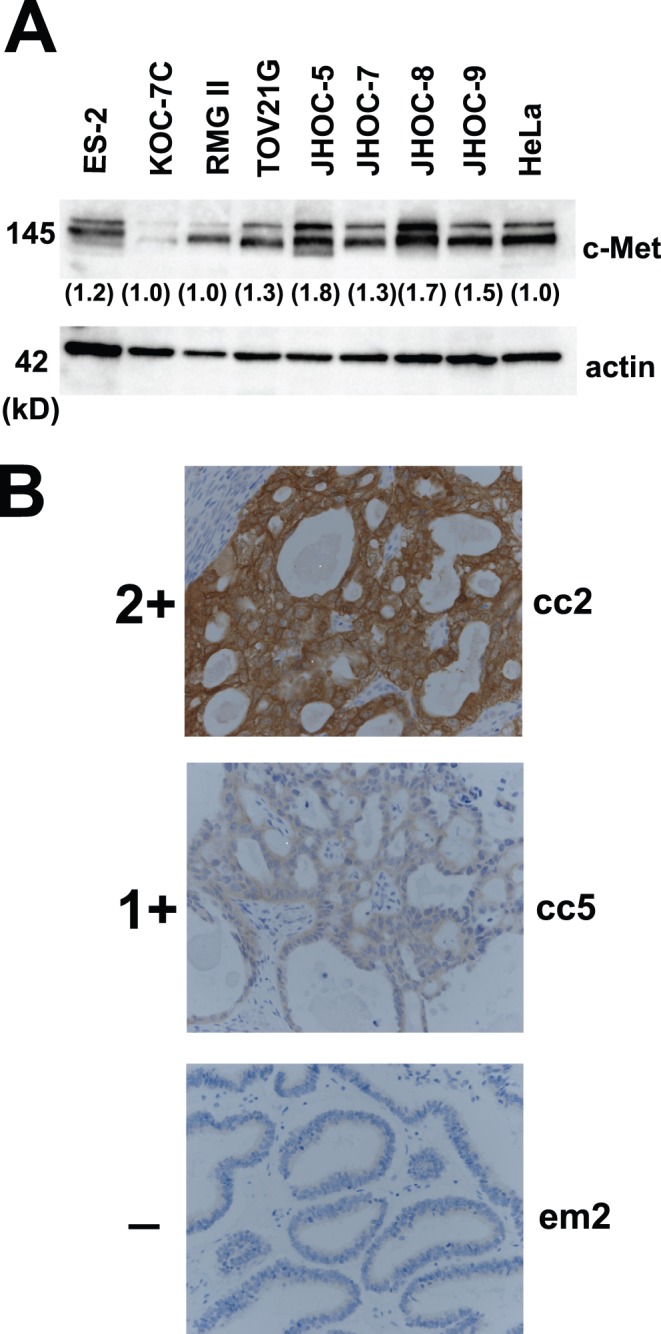
Confirmation of Met amplification at the protein level. **A.** The immunoblotting results for Met protein expression in ovarian clear cell adenocarcinoma cell lines. Two cell lines (JHOC-5 and JHOC-8) with Met gene amplification show stronger intensities. **B.** The immunostaining results of representative cases (cc2, Met-amplified ovarian clear cell adenocarcinoma (OCC) case; cc5, OCC case without Met amplification; em2, endometrioid adenocarcinoma case without Met amplification). Positive staining for c-Met antibody was further divided to 2 groups: 2+ and 1+ (see text for details).

We then confirmed amplification of the Met gene by FISH (**Fig. 3**). A centromere probe of chromosome 7 (CEP7) was used as a reference to count the amplification signals. All 4 samples that showed Met amplification by array-based CGH analysis had an increased Met/CEP 7 ratio, confirming the results of the array-based CGH analysis. We further confirmed the Met gene copy number increase by real-time quantitative PCR. Using the hTERT copy number reference as a control, we successfully demonstrated that all 4 Met-amplified samples and none of the 9 samples without Met amplification showed more than a 1.5-fold increase in DNA quantity (**Fig. 4A**). We further confirmed Met amplification in the cell lines by real-time quantitative PCR (**Fig. 4B**), which also showed good correlation with the array-based CGH analysis. To confirm that the protein level change accompanied gene alteration, we performed Western blotting using the 8 OCC cell lines (**Fig. 5A**). The total amount of Met protein was clearly increased in JHOC-5 and JHOC-8 cells, both of which demonstrated Met genome amplification. Immunostaining of 13 OCC samples also revealed overexpression of the Met protein in the gene-amplified samples (**Fig. 5B** and **Table 1**). Thus, we found Met gene amplification in 6 of the 21 OCC samples (28.5%) analyzed by array-based CGH analysis, all of which were confirmed by qPCR and protein expression analyses.

### Confirmation of AKT2 Amplification

**Figure 6:**
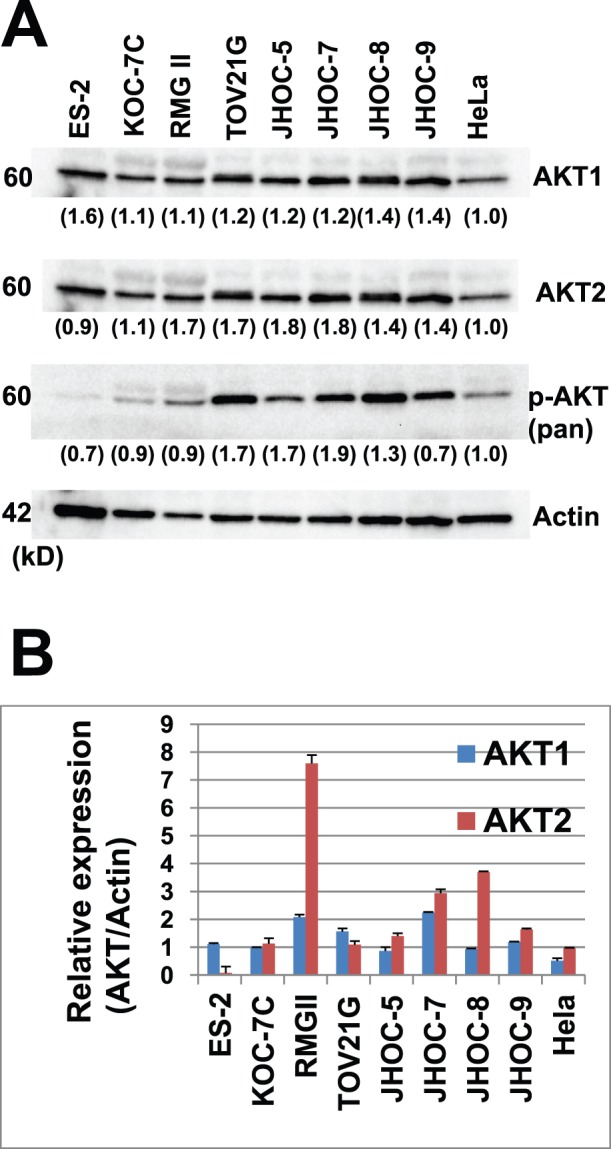
AKT1 and AKT2 expression in ovarian clear cell adenocarcinoma. **A.** Western blot analyses of protein expression using AKT antibodies in ovarian clear cell adenocarcinoma cell lines. Various intensities are observed by immunoblotting with AKT1, AKT2, and pan-AKT phosphorylated antibodies (serine 473 phosphorylated-AKT). **B.** A qRT-PCR analysis revealed relatively higher expression of AKT2 compared to AKT1 at the mRNA level.

We next confirmed the AKT2 gene copy number increase by qPCR and demonstrated that all three AKT2-amplified samples and none of the ten samples without Met amplification showed more than a 1.5-fold increase in DNA quantity (**Fig. 4C**). Amplification of the AKT2 gene was detected in two cell lines, namely, RMG-II and JHOC-8, by array-based CGH analysis (**Figs. 1** **and** **2**) and was also confirmed by real-time quantitative PCR (**Fig. 4D**). Western blotting using the 8 OCC cell lines revealed that both AKT1 and AKT2 were highly expressed in most OCC cells compared with HeLa cells, and immunoblotting using a phosphorylated-AKT (Ser473) antibody that recognizes the activated forms of all three isoforms of AKT was positive in all OCC cell lines showing AKT activation (**Fig. 6A**). To compare the relative amount of AKT isoform expression, we further performed a qRT-PCR analysis based on standard curves using plasmids encoding the PCR amplicons of AKT1 and AKT2, which were similar in length, as templates for the corresponding PCR. Interestingly, higher expression of AKT2 was observed in most OCC cell lines compared with AKT1 (**Fig. 6B**).

### Met Amplification and Overall Survival

**Figure 7:**
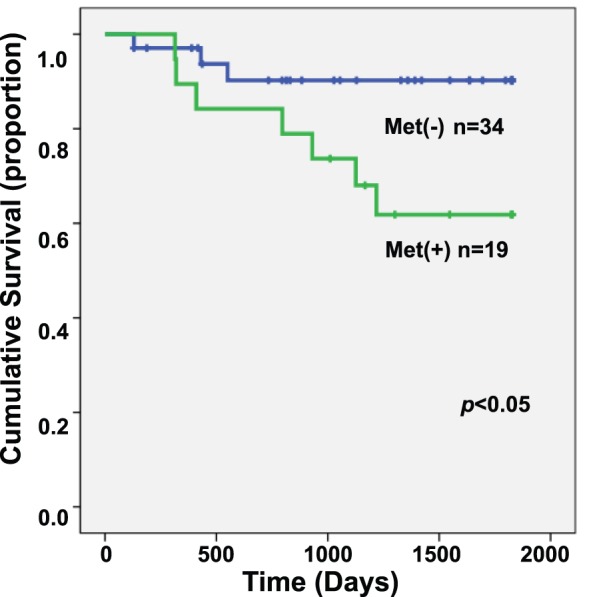
The Kaplan-Meier curve of stage 1 and 2 ovarian clear cell adenocarcinoma patients with or without Met amplification. Patients with Met amplification had a significantly worse prognosis (p<0.05).

A qPCR analysis revealed that 27 of the 73 cases had a greater than 2-fold increase in Met gene expression (37.0%). Similarly, 19 of the 53 (35.9%) stage 1 & 2 patients had Met amplification, and these patients had significantly worse overall survival compared with patients without Met amplification (p = 0.037) (**Fig. 7**). We then analyzed 73 OCC samples to calculate the exact rate of AKT2 amplification. However, in total, only 6 of the 73 cases had a greater than 2-fold increase in AKT2 gene expression (8.2%) by qPCR, and no significant difference in survival was detected. We therefore focused on Met in further analyses.

### Met Knockdown Experiments *in vitro*

**Figure 8:**
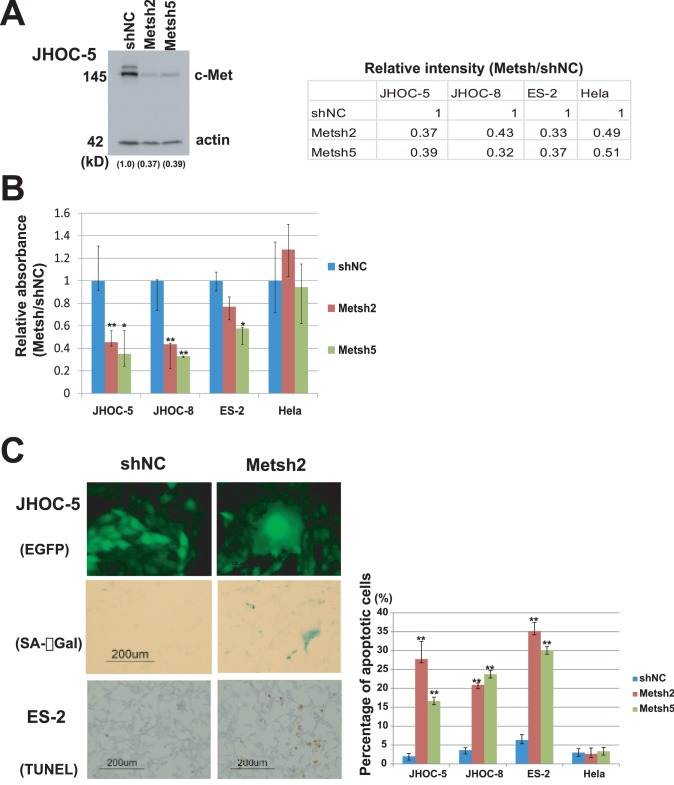
Knockdown of Met in ovarian clear cell adenocarcinoma cell lines. A. Confirmation of knockdown by Western blotting. Signal intensity ratio (Met/actin) are shown in numbers. B. Cell viability assay after transfection using retroviral vectors encoding short-hairpin RNAs targeting Met (Metsh2 and Metsh5) or control, shNC, in ovarian clear cell adenocarcinoma (OCC) cell lines. A decrease in cell viability in OCC cell lines with Met amplification (JHOC-5 and JHOC-8) is evident compared to ES-2 (OCC cell without Met amplification) or control HeLa cells. *p<0.05, ** p<0.01** C.** Morphologically, Met-amplified cells (JHOC-5) expressing Metsh and enhanced green fluorescent protein (EGFP) became larger and were positively stained with SA-βGal (a senescence marker) compared with control. The number of apoptotic cells with nuclear staining in TdT-mediated dUTP-biotin nick end labeling (TUNEL) assay also significantly increased as a result of Met knockdown in cells with Met amplification (JHOC-5 and JHOC-8) or without Met amplification (ES-2), compared with controls.

Met knockdown resulted in a large decline of cell proliferation and survival in Met-amplified OCC cell lines (**Fig. 8**). We confirmed Met knockdown at the protein level by Western blotting (**Fig. 8A**). Met knockdown in JHOC-5 and JHOC-8 cells by two independent shRNAs resulted in a significant decrease in the number of viable cells in the MTT assay compared with control shRNA (**Fig. 8B**). This was further confirmed to be the result of significantly larger numbers of cells undergoing apoptosis in OCC cell lines, and positive senescence marker staining only in Met-amplified OCC cell lines; JHOC-5 and JHOC-8 (**Fig. 8C**), suggesting that an active Met pathway was necessary for both cell survival and proliferation. Met knockdown in ES-2 cells, an OCC cell line without Met amplification, showed a partially significant decrease in cell numbers by the cell viability assay using MTT assay compared with control shRNA (**Fig. 8B**), and this was mostly due to increased apoptosis in these cells (**Fig. 8C**). Met knockdown in HeLa cells did not have a significant impact on the results of the MTT, TUNEL, and senescence assays (**Fig. 8**).

## Discussion

In this study, we successfully detected Met amplification by array-based CGH analysis in 6 of the 21 OCC samples, and the final amplification rate was 37.0% when 73 cases were evaluated. Using similar methods, previous studies have not detected such frequent amplification of a specific gene. The reason for the discrepancy remains unclear. However, we used DNA extracted from paraffin sections, in which estimation of the tumor proportion by histological analyses is easier, and DNA was extracted from regions with a tumor proportion greater than 70%. Regional and ethnic differences of the patients may have also played a role. Yamamoto et al reported Met amplification in 28% of Japanese OCC cases [13], and Rhaman et al demonstrated that ZNF217 at chromosome 20q13.2 was amplified in 20% of their OCC patients [14]. Our array-based CGH data also showed rather frequent amplification of various genes (**Table S1**), but Met was the most frequently amplified, confirming Yamamoto’s findings. We also demonstrated the frequent amplification of AKT2, which was identified in 5/21 samples using array-based CGH analysis and 7.5% of the 73 OCC samples using qPCR. Our array-based CGH data also revealed ZNF217 amplification in 2 cell lines (RMG-II and JHOC-9) and Her2 amplification in one cell line (RMG-II) (**Fig. S2**), but neither gene was included in **Table S1** because of the lower frequency. Most recently, Yamamoto et al also reported amplification of actinin 4 at chromosome 19 13.2 [18]. Although several genes at this region were included in our list (**Table S1**), actinin 4 was also not significantly amplified in our cases. In addition, our OCC cases were primarily associated with endometriosis, which is typically prominent in Japanese cases [1], [19] and was histologically confirmed in the majority of our cases (**Table 1**). Thus, the association with endometriosis may have been related to the frequency of gene amplification. We further confirmed Met amplification using various methods such as qPCR, immunoblotting, and immunohistochemistry. All of these results showed good correlation, and we therefore suggest that Met amplification may be one of the key molecular events in OCC development, particularly in Japanese cases. Most recently, we reported that Met amplification was frequently observed in the tumor samples of an Fe-NTA-induced rat renal carcinogenesis model [4]. We propose that oxidative stress due to excess iron deposition is the primary cause of carcinogenesis in endometriosis-associated ovarian clear cell carcinomas [2], [20]. It is then interesting that a key molecular event, Met amplification, is commonly observed in both human OCC and the Fe-NTA-induced animal model. This emphasizes the importance of an underlying biological and molecular mechanism of iron-induced carcinogenesis.

In this study, we also observed amplification of AKT2, one of the three isoforms of the AKT kinase family [21]. Although previously reported in ovarian serous neoplasms [22], our study is the first to report AKT2 amplification in clear cell adenocarcinoma. PI3K is one of the downstream effectors recently shown to be mutated in more than 30% of OCC cases [6]. AKT is a further downstream component of the Met/PI3K signaling pathway [23]. Considered alongside our data, this suggests that activation of the Met/PI3K/AKT2 pathway may have an important role in OCC carcinogenesis. mTOR, another component of the Met/PI3K/AKT2 pathway downstream of PI3K, inhibits p53 via increased Mdm2 translation [24], and p53 is known to induce both apoptosis and senescence [25]. Because Met knockdown in OCC cell lines with Met amplification showed both increased apoptosis and senescence *in vitro* (**Fig. 8**), we believe that Met-amplified OCC cells primarily depend on activation of the Met/PI3K/AKT pathway for cell proliferation and survival.

Met gene amplification was first reported in a gastric carcinoma cell line [26], followed by a proportion of gastric and esophageal adenocarcinomas [27]–[29], non-small cell carcinoma of the lung [30], [31], colorectal adenocarcinoma [32], and squamous cell carcinoma of the head and neck [33]. Met amplification is usually accompanied by overexpression of the Met protein [34], and studies have been conducted to elucidate the biological significance of Met overexpression with or without amplification, with mostly similar conclusions: tumors with Met overexpression have a worse prognosis [30], [34]–[39]. Furthermore, recent reports have proposed that the growth and survival of Met-amplified tumor cells are fully dependent on Met and explained this as an example of ‘oncogene addiction’ [40], [41]. In our study, OCC cell lines with Met amplification were significantly dependent on Met for cell growth and survival. We therefore believe that OCC tumor cells are also ‘addicted’ to Met. Previous studies have demonstrated inactivation of the Met pathway by specific small inhibitors [42], [43], which are particularly effective in the treatment of ‘Met oncogene-addicted’ carcinomas [40], and we suggest that OCC is another optimal target for Met-targeting therapies.

In conclusion, we demonstrated frequent genomic amplification of the Met gene by comprehensively analyzing the total genome, showing that Met was the most frequently amplified gene in Japanese OCC samples. Furthermore, we also detected the amplification of AKT2, which is a downstream component of the Met/PI3K signaling pathway. Targeted therapy inhibiting the Met/PI3K/AKT pathway may be promising for OCC treatment.

## Supporting Information

Figure S1 **Frequency distribution result of genomic alterations in each chromosome of array-based comparative genome hybridization analysis.** Relative frequencies of genomic loss (green bar) and gain (red bar) for 13 primary ovarian clear cell adenocarcinoma tissue samples are plotted at each chromosomal position. Regions of genomic alteration in a single profile were identified using the Z-score statistical algorithm.
(PDF)

Figure S2 **Gene views of chromosomes 20 and 17 of the genomic changes observed by array-based CGH analysis in the 21 ovarian clear cell adenocarcinoma samples.** Low peaks within 1 Mb are observed in 2/21 samples at the genomic region encoding ZNF217 (upper row). The amplified region including Her2 is also shown (lower row).
(PDF)

Table S1 Frequently amplified regions of the chromosome and included genes detected by array-based comparative genomic hybridization.
(XLSX)
