# Correction: Met Is the Most Frequently Amplified Gene in Endometriosis-Associated Ovarian Clear Cell Adenocarcinoma and Correlates with Worsened Prognosis

**DOI:** 10.1371/journal.pone.0342829

**Published:** 2026-02-17

**Authors:** The PLOS One Editors

Following publication of this article [1], the following errors were identified in Figs 3 and 6:

- In Fig 3B, the y axis label is erroneously omitted.
- In Fig 6A, the AKT1 panel is incorrect and is a duplicate of the AKT2 panel. The corresponding author stated that the incorrect AKT1 panel was used following an error made during revision of the figure.
- In Fig 6A, the corresponding Actin blots are omitted for AKT1 and p-AKT (Pan) proteins and AKT1 and p-AKT (Pan) are incorrectly quantified against a single Actin blot, which is the loading control for the AKT2 blot.

The revised Fig 3 is included with this Correction.

The corresponding author has provided a revised Fig 6 in which the incorrect AKT1 panel is replaced with the correct image from the original experiments and the corresponding Actin blots from the original experiments are also included. The revised Fig 6 also includes updated AKT/Actin ratios calculated in ImageJ for each AKT protein quantified against the correct corresponding Actin blot.

During editorial follow-up, the corresponding author provided the following additional information:

- The Actin panels in Figs 5A and 6A are intentionally reused because the same membrane was used for immunoblotting of AKT2, c-Met, and Actin, with the antibodies stripped and re-hybridized.
- The original quantitative data underlying Figs 3, 4, 5A, 6A, 7, and 8 are unavailable. The updated quantitative data included in the revised Fig 6A are unavailable as this was copied directly from ImageJ. The original images underlying Figs 5A and 6A and image data from a repeat immunoblot of AKT2 are available and included in S1 File. Quantitative data underlying Fig 6B are available and shared in S2 File.

**Fig 3:**
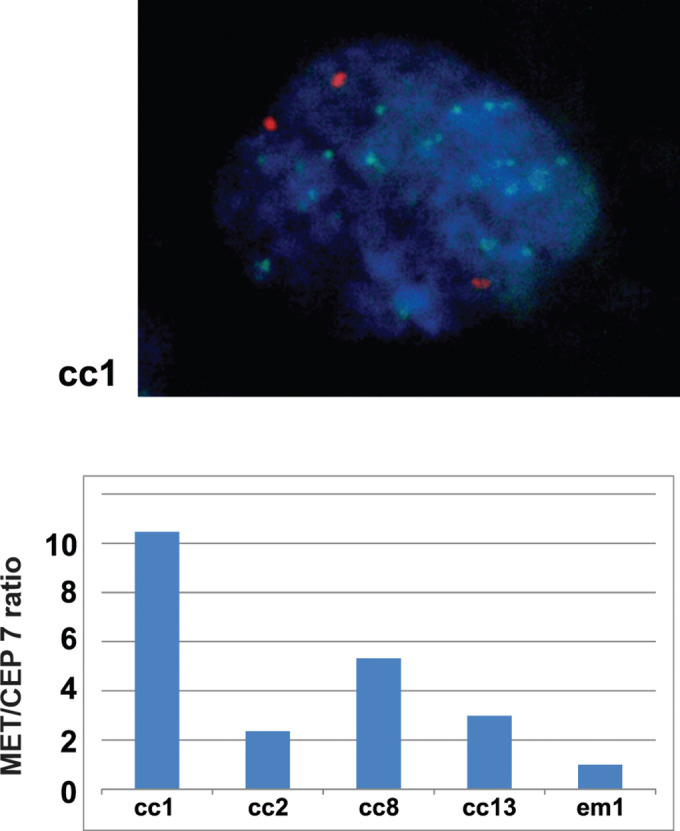
FISH analysis for confirmation of Met amplification. A representative nucleus of a Met-amplified cell (cc1) is shown in the upper figure (Green: Met probe, Orange: CEP 7; centromere 7 probe, Blue; DAPI). The lower graph shows the FISH signal number (MET/CEP 7 ratio) of the 4 Met-amplified ovarian clear cell adenocarcinoma samples (cc1, cc2, cc8, cc13) and an endometrioid adenocarcinoma case (em1) without Met amplification. A total of 60 cells were counted for each sample, average numbers (Met: CEP7) were as follows; cc1(18 : 2.0), cc2(4.4 : 2.1), cc8(10 : 2.2), cc13(4.6/1.7), em1(1.7/1.9). All values were then normalized with that of em1 as 1.0.

**Fig 6:**
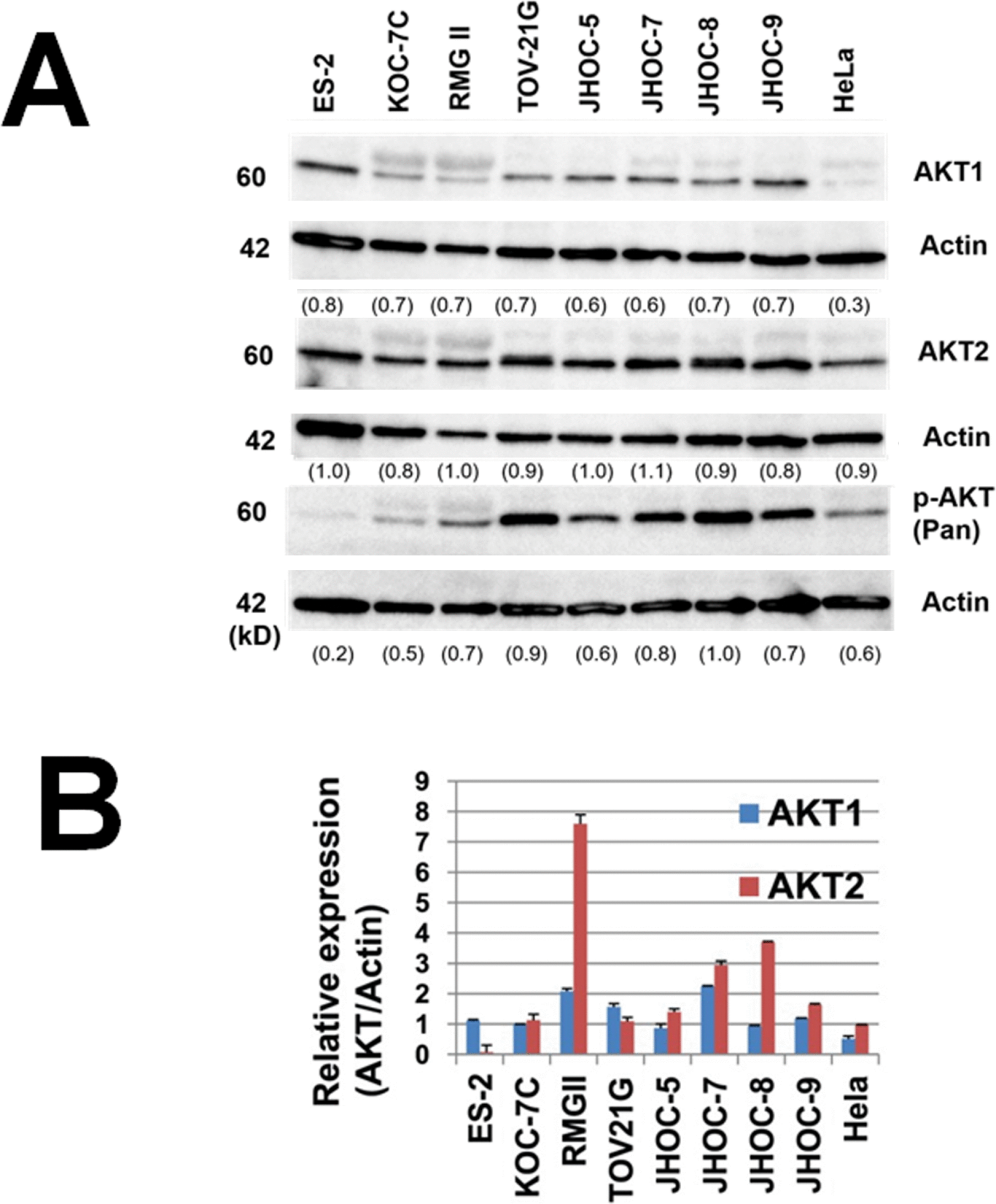
AKT1 and AKT2 expression in ovarian clear cell adenocarcinoma. **A.** Western blot analyses of protein expression using AKT antibodies in ovarian clear cell adenocarcinoma cell lines. Various intensities are observed by immunoblotting with AKT1, AKT2, and pan-AKT phosphorylated antibodies (serine 473 phosphorylated-AKT). **B.** A qRT-PCR analysis revealed relatively higher expression of AKT2 compared to AKT1 at the mRNA level.

## Supporting information

S1 File Original images underlying Figs 5 and 6. Underlying image data for AKT1, C-Met and AKT2, and pAKT serine 473. Underlying image data for a repeat immunoblot of AKT2 from a different experiment completed during the same period.
(ZIP)

S2 File Quantitative data underlying Fig 6B. qRT-PCR analysis for AKT1 and AKT2.
(XLSX)
