# Supplementary figures and images for: Met Is the Most Frequently Amplified Gene in Endometriosis-Associated Ovarian Clear Cell Adenocarcinoma and Correlates with Worsened Prognosis
Source: PLoS One. 2013 Mar 4;8(3):e57724. doi: 10.1371/journal.pone.0057724 (PMC3587638; doi:10.1371/journal.pone.0057724)

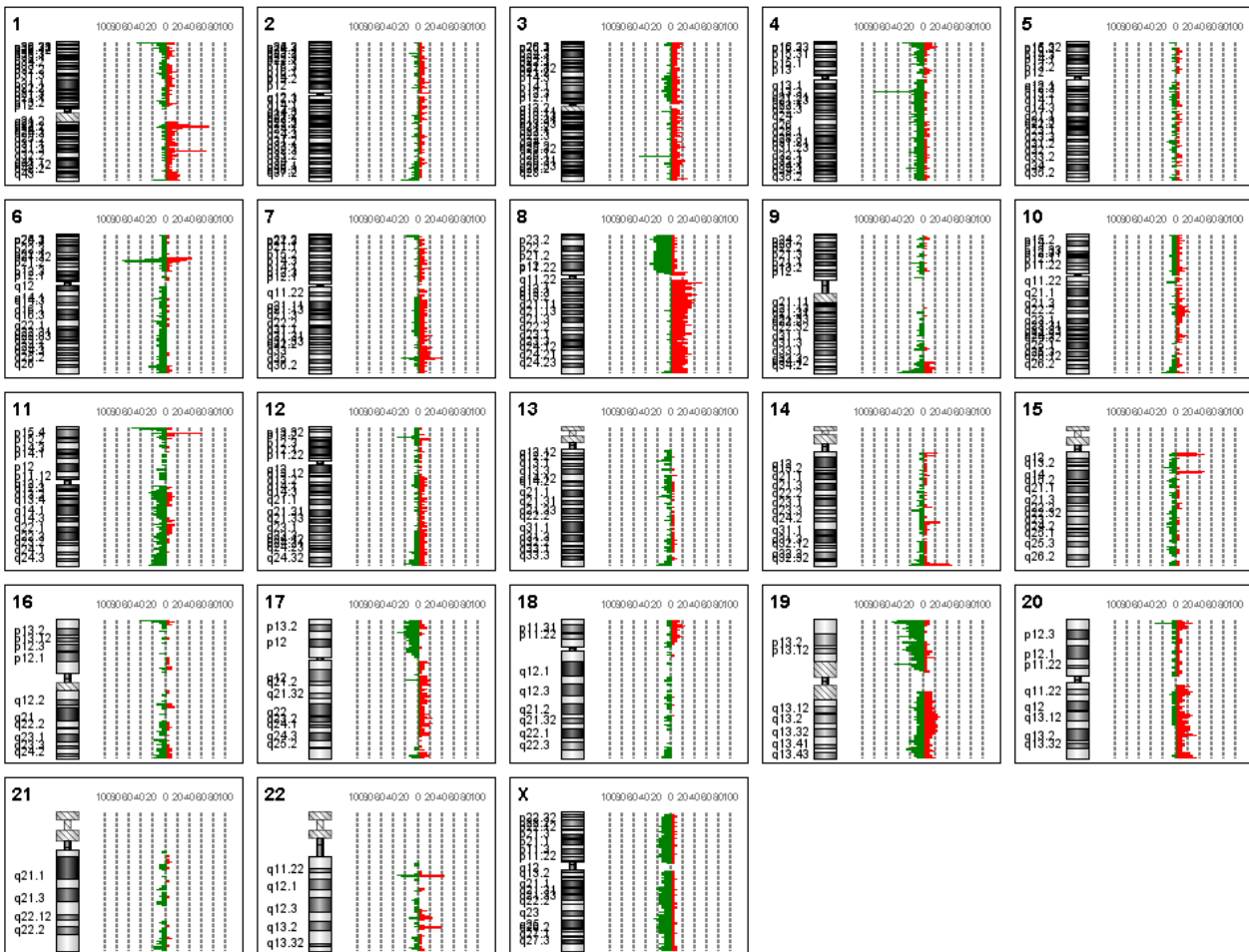

Supplement: Figure S1 — Frequency distribution result of genomic alterations in each chromosome of array-based comparative genome hybridization analysis. Relative frequencies of genomic loss (green bar) and gain (red bar) for 13 primary ovarian clear cell adenocarcinoma tissue samples are plotted at each chromosomal position. Regions of genomic alteration in a single profile were identified using the Z-score statistical algorithm. (PDF) [file pone.0057724.s001.pdf]

## Chromosome 20

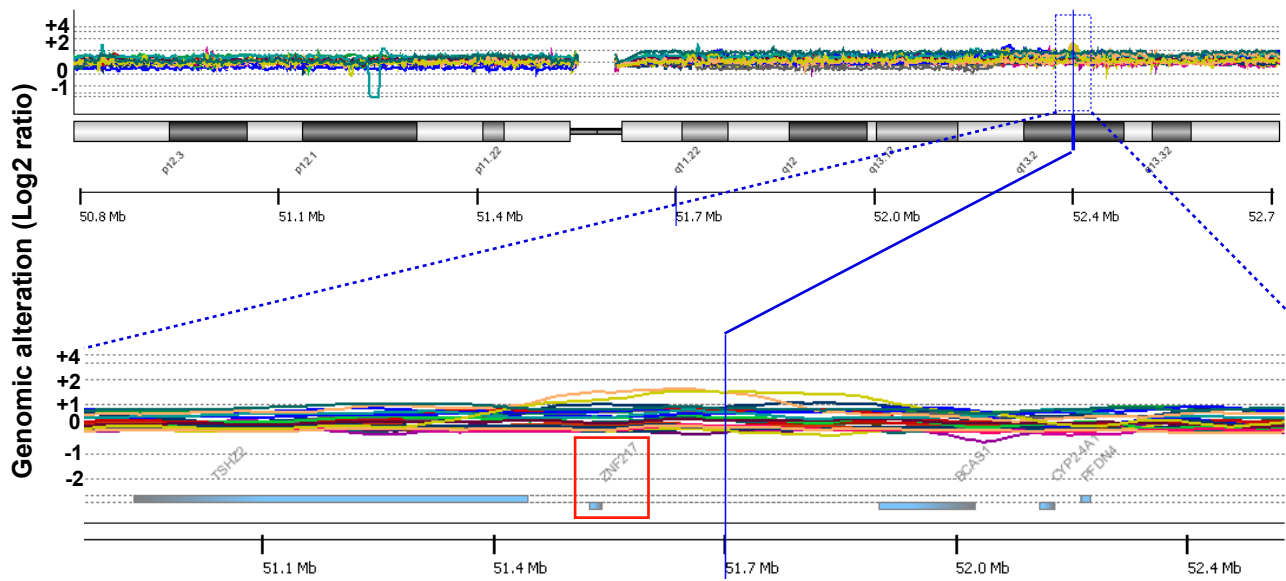

## Chromosome 17

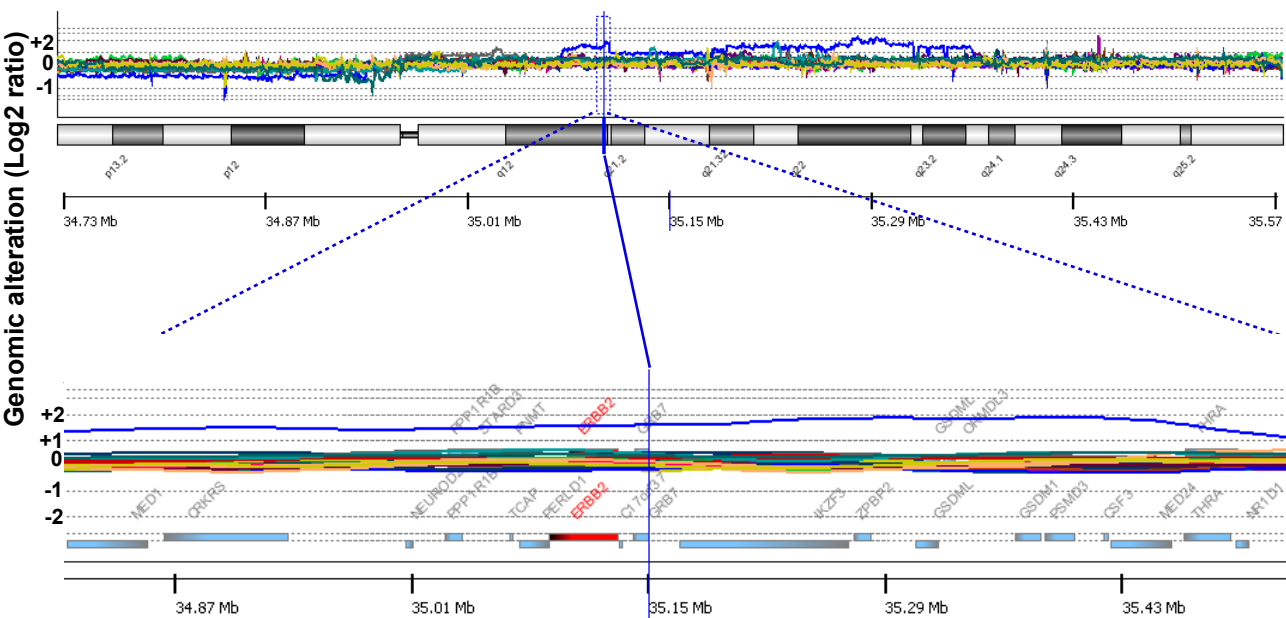

Supplement: Figure S2 — Gene views of chromosomes 20 and 17 of the genomic changes observed by array-based CGH analysis in the 21 ovarian clear cell adenocarcinoma samples. Low peaks within 1 Mb are observed in 2/21 samples at the genomic region encoding ZNF217 (upper row). The amplified region including Her2 is also shown (lower row). (PDF) [file pone.0057724.s002.pdf]
