# Supplementary figures and images for: Correction: Met Is the Most Frequently Amplified Gene in Endometriosis-Associated Ovarian Clear Cell Adenocarcinoma and Correlates with Worsened Prognosis
Source: PLoS One. 2026 Feb 17;21(2):e0342829. doi: 10.1371/journal.pone.0342829 (PMC12912569; doi:10.1371/journal.pone.0342829)

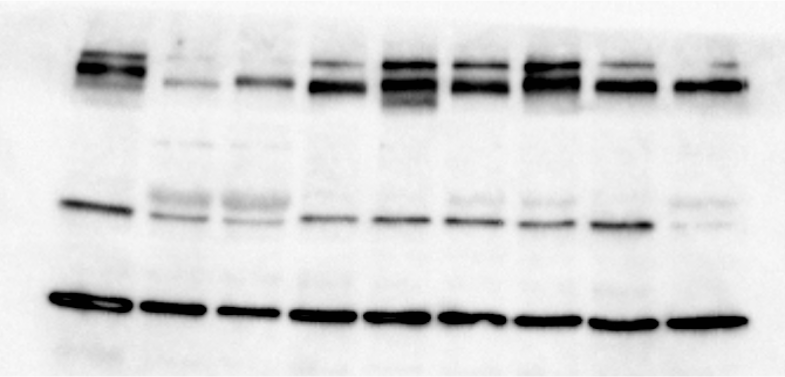

Supplement: S1 File — Underlying image data for AKT1, C-Met and AKT2, and pAKT serine 473. Underlying image data for a repeat immunoblot of AKT2 from a different experiment completed during the same period. (ZIP) [file pone.0342829.s001.zip › S1 File/AKT1.png]

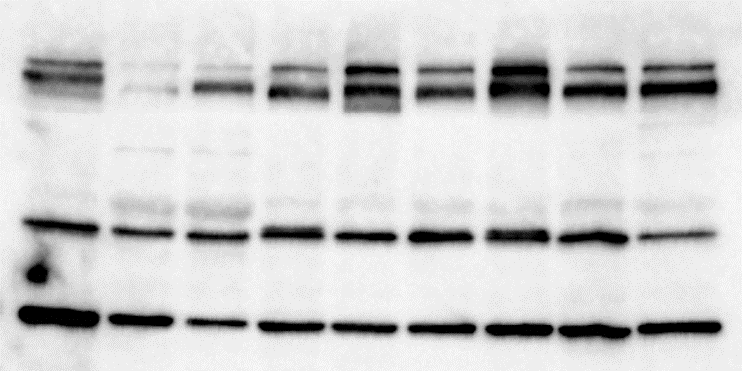

Supplement: S1 File — Underlying image data for AKT1, C-Met and AKT2, and pAKT serine 473. Underlying image data for a repeat immunoblot of AKT2 from a different experiment completed during the same period. (ZIP) [file pone.0342829.s001.zip › S1 File/C-Met and AKT2.png]

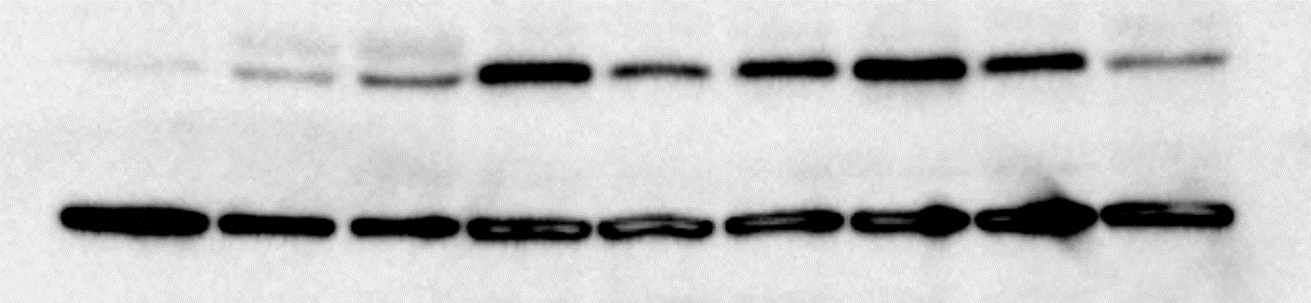

Supplement: S1 File — Underlying image data for AKT1, C-Met and AKT2, and pAKT serine 473. Underlying image data for a repeat immunoblot of AKT2 from a different experiment completed during the same period. (ZIP) [file pone.0342829.s001.zip › S1 File/pAKT serine473.png]
